# A Case of Strongyloidiasis: An Immigrant Healthcare Worker Presenting with Fatigue and Weight Loss

**DOI:** 10.1155/2017/6718284

**Published:** 2017-06-28

**Authors:** Tarundeep Grewal, Heela Azizi, Alexa Kahn, Zaid Shakir, Sahar Takkouche, Khin N. Aung, William Lois, Muhammad Hasan

**Affiliations:** ^1^Kingsbrook Jewish Medical Center, Brooklyn, NY, USA; ^2^American University of Antigua College of Medicine, Coolidge, Antigua and Barbuda

## Abstract

**Background:**

* Strongyloides stercoralis* is an intestinal nematode parasite classified as a soil-transmitted helminth, endemic in tropical and subtropical regions.* Strongyloides stercoralis *can remain dormant for decades after the initial infection.

**Case:**

We describe a patient who was diagnosed with* Strongyloides stercoralis* infection three weeks after a left inguinal hernia repair and discuss approaches to prevention, diagnosis, and treatment.

**Conclusions:**

Physicians in the United States often miss opportunities to identify patients with chronic strongyloidiasis. Symptoms may be vague and screening tests have limitations. We review current strategies for diagnosis and treatment of chronic intestinal strongyloidiasis in immigrant patients who have significant travel history to tropical regions and discuss the clinical features and management of the infection.

## 1. Introduction

Infecting more than 100 million people around the world,* Strongyloides* is a parasite most commonly found in immigrants. As many as 50% of patients remain asymptomatic and can survive decades undiagnosed [1]. These parasites can cause infections that result in serious illness and even death [2]. A 20-month study conducted in Honduras identified predisposing factors associated with* Strongyloides* infection including transplants, malignancy, and asthma treated with steroids and traveling to endemic countries. These parasites are prevalent in the Caribbean, Latin America, Europe, Asia, and sub-Saharan Africa [3]. As determined in this study, not all patients were found to have eosinophilia in peripheral blood, a finding normally linked to* Strongyloides*. Most commonly, presentations of the infection include nausea, loss of appetite, weight loss, and diarrhea. A normal eosinophil count is correlated with a poor prognosis for the patient due to clinician's lack of suspicion of an infection [4]. Immunosuppressed patients, with chronic infection of* Strongyloides stercoralis*, are at risk for hyperinfection. Hyperinfection is an overproliferation of larvae, often disseminating to the brain, lungs, and liver. This uncontrolled process can cause sepsis in the patient, with an initial presentation of wheezing, hemoptysis, and fever [5]. When it comes to tracing illnesses with such associated symptoms, most physicians often overlook inquiring about patients' travel history. This consequently leads to an incomplete health assessment and can result in a delayed diagnosis or misdiagnosis.

## 2. Case Presentation

A 74-year-old female retired healthcare worker presented with abdominal pain and vomiting following a left inguinal hernia repair performed 7 days priorly. Symptoms began two days after her surgical procedure. Patient had a seven out of ten nonradiating, diffuse abdominal pain localized to the epigastric region described as burning in nature. It was accompanied by fever, nausea, and postprandial vomiting, which was clear and mucoid in nature, sometimes containing food particles. She did not have bowel movements since her inguinal hernia repair. Upon further inquiry, the patient mentioned that she had a decrease in appetite for all types of food for the past 6 months and began limiting her intake to liquids because they were easier to tolerate. The patient also noticed changes in stool caliber and noted an unintentional weight loss of 25 pounds in a one-month span. The patient stated that laboratory tests were unremarkable. A stool exam was not ordered since patient had no complaints of diarrhea. She had an esophagogastroduodenoscopy performed four months priorly due to similar symptoms and the results were essentially normal. The patient has a history of osteoarthritis. Surgical history includes umbilical hernia, a hysterectomy in 2012, and a left inguinal hernia in 2015. Family history includes diabetes mellitus and breast cancer. Patient is a retired home healthcare worker who resides in Grenada six months out of the year and frequently visits the United States. Patient has no other significant medical conditions and denies use of tobacco, alcohol, or illicit drugs. In review of systems, the patient complains of feeling dizzy and experienced a severe bout of malaise beginning around the time she began to lose weight. However, no signs of fever, pruritus, or erythema were noted. The patient was asked about any distinctive food choices or habits and she states that she plants and consumes her own vegetables in rural Grenada.

Upon examination, the patient was an elderly female of the appropriately stated age; she appears apathetic and fatigued. The patient was malnourished and in mild distress with vital signs reading: blood pressure of 135/90 mmHg, a pulse of 110 bpm, a temperature of 99.8°F, and a respiratory rate of 18 breaths per minute. Lungs were clear to auscultation bilaterally and normal S1 and S2 sounds were heard with regular rate and rhythm. The abdomen was soft, nondistended, tender to light, and deep palpation in the umbilical area but no masses or organomegaly was felt. Bowel sounds were normoactive in all 4 quadrants and a surgical scar from umbilical hernia was noted. Patient's left inguinal surgical site was tender to touch with mild swelling and no drainage.

The abdominal X-ray and CT were ordered and suggested evidence of ileus and dilated stomach with small bowel distended up to 4.5 cm diameter without identifiable obstruction, respectively. No organomegaly or lymphadenopathy was noted. Upper GI follow-through was performed and impressions identified jejunal mural abnormality with thickening and loss of normal mucosal pattern, suggestive of broad region of infection or inflammation. Laboratory examination revealed an unremarkable absolute eosinophil count and blood percentage (Table 1).

Patient presented with an elevated white blood cell count of 11,000/mm^3^ which rose to 16,000 mm^3^, following days of admission which is related to the infected surgical site (Table 1). Tumor markers for CA-19-9, CEA, and CA-125 were also measured and were within normal limits to rule out malignancy.

**Figure 1:**
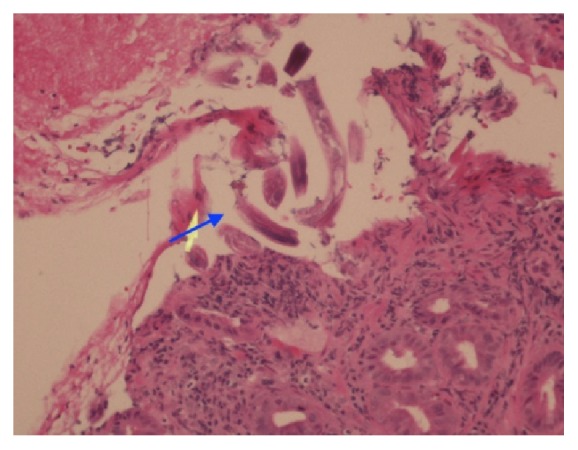
Haematoxylin and eosin (H&E) stain, gastric mucosa nonfundic type, pointing to fragments of* Strongyloides stercoralis* under 200x (blue arrow).

**Figure 2:**
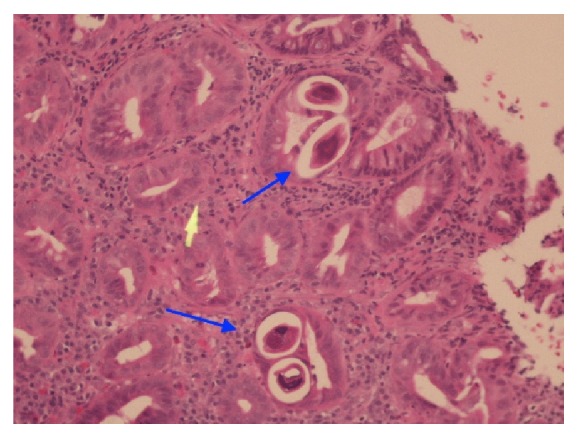
Haematoxylin and eosin (H&E) stain, gastric mucosa nonfundic type, and cross section view of* Strongyloides stercoralis* under 200x (blue arrows).

The patient was admitted for possible small bowel obstruction which was worked up with conservative treatment. Patient was placed on a regimen of nothing by mouth (NPO), lactated ringer 1000 mL intravenously at 125 mL/hr, NGT tube for intermittent decompression, and ketorolac 20 mg subcutaneously every 6 hours for pain. Another esophagogastroduodenoscopy was performed and the impression revealed duodenal mucosal thickening and diffuse redness. Biopsies were also taken: one sample from the antrum of the stomach and three samples from the duodenum which were submitted in formalin with the largest fragment measured up to 0.5 × 0.3 × 0.2 cm. Microscopic examination samples in the nonfundic type of gastric mucosal biopsy showed numerous parasites in the lumen of the glands (Figures 1 and 2). Parasite sections were noted in cross-sectional views and in other areas showing in long sections, which were identified as* Strongyloides stercoralis*. Eosinophils were identified in the infiltrate at 200x to 600x magnification, but a count was not taken. The diagnosis was confirmed with a stool ova and parasite, revealing rhabditiform larvae, which typically measure 200 to 300 microns by 10 to 20 microns [6].

## 3. Treatment

Patients with suspected strongyloidiasis often require serologic testing including ELISA to detect IgG to filariform larval antigen, or stool culture. Stool ova may be used but, for the screening test to reach a sensitivity of greater than 90%, a greater number of samples are required. Therefore, serologic testing is more widely available and used to confirm the diagnosis [2, 5]. The patient received ivermectin 9 mg taken orally, once per day for two days. The gold standard treatment for uncomplicated strongyloidiasis is ivermectin as two single 200 mg/kg doses administered on two consecutive days. Albendazole, 400 mg PO twice daily for three to seven days, is an alternative but has an efficacy of 78% compared to ivermectin with 100% [7]. Eosinophil and serologic tests were ordered a month after treatment to verify cure; results are pending. Patients with hyperinfection syndrome often receive a typical dose of ivermectin 200 mcg/kg daily for at least five to seven days or a combined ivermectin with albendazole dose until the symptoms have resolved. Furthermore, patients with unresolving hyperinfection should be suspected for human T-lymphotropic virus type I infection (HTVL-1) [8].

## 4. Discussion/Conclusion

*Strongyloides* infections have a wide range of presenting symptoms and vary in location throughout the gastrointestinal tract. In an endoscopic pathologic study of 6 patients, 4 cases involved the small bowel, 2 cases in the colon, and one case in the stomach [9]. These infections are predominantly seen in migrants and returning travelers from endemic areas in the tropics and subtropics [5]. Since* S. stercoralis* is distributed amongst the soil found in these tropical areas, a thorough history is necessary to determine the patient's potential exposure to the parasite [10]. In travelers or migrants, helminthic infection is the commonest identifiable cause of eosinophilia. In many intestinal parasitic infections, eosinophilia can be transient and is associated with the tissue migratory phase of the infection [11]. Strongyloidiasis is known as a cosmopolitan neglected disease whose complications are strongly associated with alcoholism, organ transplants, HTLV-1 virus, and immunosuppression in general [3].

*Strongyloides stercoralis* is frequently underdiagnosed due to the low sensitivity of common fecal diagnostic methods used in clinical laboratories [12]. This leads to the prevalence being low as 3.3% in areas such as Grenada [13]. There is a misconception that the overall prevalence of* S. stercoralis* is low, in part because of the low sensitivity of many of the fecal diagnostic tools used in clinical laboratories. Ultimately, the low sensitivity of diagnostic tools leads to underdiagnosis of* Strongyloides *[12]. One explanation for this is the low larval output in patient stool samples. The ELISA is another diagnostic method; however, it is not always accessible and this test does not differentiate between previous infection and other helminthic infections, making a definitive diagnosis difficult. Aspirate materials and pathological tissue sampling will determine the diagnosis. In immunocompromised patients, early diagnosis is imperative to preventing hyperinfection and systemic dissemination [14]. Therefore, the need for thorough travel history is important because if strongyloidiasis is diagnosed early, it is easily treatable with oral antihelminthic drugs. The preferred treatment for strongyloidiasis is ivermectin, and it should be initiated even in the absence of symptoms, to prevent parasite dissemination and hyperinfection [15].

In conclusion, immigrant patients from endemic areas who show clinical signs and symptoms of helminthic infection, such as nausea, fatigue, and unintentional weight loss should have a thorough history in order to make early diagnosis and prevent further complications, such as superinfections in the immunocompromised population. Physicians in the US should consider testing for the parasite in any immigrant from endemic countries to aid in reducing the number of cases that go undiagnosed yearly.

## Figures and Tables

**Table 1:** Patient's laboratory examination results (hematology).

	Feb 22-17	Feb 27-17	Mar 02-17
Leukocyte count (4.8–10.8) × 10^3^/L	11.0	13.5	16.2
Neutrophil (40–60%)	71.8	76.5	78.4
Eosinophil (1–4%)	1.4	1.0	1.0
Absolute eosinophil count (0.1–0.4) × 10^3^/L	0.2	0.1	0.2
